# Super‐Robust Telecommunications Enabled by Topological Half‐Supermodes

**DOI:** 10.1002/advs.202515157

**Published:** 2026-01-09

**Authors:** Rui Zhou, Xintong Shi, Hai Lin, Yan Ren, Hang Liu, Zihao Yu, Jing Jin, Zhihao Lan, Menglin L. N. Chen

**Affiliations:** ^1^ College of Physical Science and Technology Central China Normal University Wuhan Hubei China; ^2^ Department of Electrical and Electronic Engineering The Hong Kong Polytechnic University Hong Kong China; ^3^ Department of Electronic and Electrical Engineering University College London London UK; ^4^ Shenzhen Research Institute The Hong Kong Polytechnic University Shenzhen China

**Keywords:** seamless integration, super‐robust telecommunications, topological half‐supermodes, ultra‐compact, valley‐ridge hybrid waveguides

## Abstract

Topological photonics offer transformative potential for robust integrated waveguide devices due to their backscattering‐immune properties. However, their integration faces two fundamental challenges: mode symmetry mismatch with conventional waveguides and prohibitive dimensions. We successfully overcome these two critical challenges by introducing a novel valley‐ridge gap waveguide based on topological half‐supermode engineering. By strategically hybridizing ridge waveguide modes and valley kink states, we create an exotic odd‐symmetric supermode enabling robust propagation and ultra‐compact operation. The further implementation of a perfect electric conductor boundary halves the lateral dimensions while eliminating radiation loss. Crucially, our proposed valley–ridge interface achieves direct transverse electric mode matching with standard waveguides without transition structures, enabling seamless integration. Experimental results demonstrate reflection losses lower than −15 dB in realistic telecommunication scenarios with super‐robust signal propagation through sharp bends. This work innovatively conceptualizes topological half‐supermodes and pioneers their practical applications for integrated waveguide devices, establishing a completely new waveguide class that uniquely combines robust backscattering immunity with deep subwavelength compactness.

## Graphical abstract

Through topological half‐supermode engineering, we realize a valley‐ridge gap waveguide that is directly compatible with conventional waveguides and features topological protection. This design halves the lateral size via a PEC boundary, enabling direct TE₁₀‐mode matching and robust bend‐immune transmission. We thus establish a new class of compact, directly integrable mmWave waveguides.

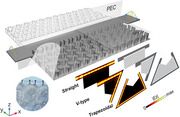

## 1 Introduction

Waveguiding devices [1, 2] for advanced wireless communications systems form essential components of modern high‐frequency systems, enabling critical applications from 5G/6G networks to satellite communications and radar systems [3, 4, 5]. Among these, rectangular waveguides represent the gold standard due to their exceptional shielding, low signal attenuation, and high‐power handling capability [6, 7]. However, their fully enclosed metallic structures require precision fabrication and result in imposing rigidity, limiting their deployment in compact and robust communications platforms.

The emergence of topological photonics [8, 9, 10, 11], particularly valley Hall phases [12, 13, 14, 15, 16, 17, 18], has introduced new opportunities for backscattering‐immune waveguiding against fabrication imperfections and sharp bends. Researchers have leveraged valley edge states for signal transmission in various integrated topological systems, such as metasurfaces [19, 20, 21], substrate‐integrated waveguides [22, 23], parallel‐plate metallic waveguides [24, 25, 26, 27], and silicon‐based waveguides [28, 29, 30]. However, their fundamental incompatibility with conventional waveguide modes presents persistent challenges. Topological edge states are typically tightly confined at domain walls with exponentially narrow mode widths, making them incompatible with standard waveguiding modes. Although three‐layer heterostructures [31, 32, 33, 34, 35, 36, 37] have introduced adjustable mode width in topological waveguides, the supported modes possess unconventional profiles that demand transition structures to match with transverse electromagnetic (TEM) or transverse electric (TE_10_) modes. Moreover, valley edge states with convex dispersion present odd‐symmetric modes that are incompatible with the even‐symmetric fundamental modes of conventional waveguides [38, 39, 40, 41, 42]. Current solutions to address this symmetry mismatch rely on engineered transition elements, such as asymmetric slot lines [38, 41] generating compensating anti‐phase fields. Alternative approaches have exploited even‐symmetric valley edge states but with concave dispersion [37, 39, 40, 42] to achieve compatibility with conventional waveguide modes. Nevertheless, all these solutions remain constrained by narrowband operation and stringent fabrication tolerances. Besides, valley photonic crystals (VPCs) require domain‐wall structures to create their bandgap properties, leading to increased lateral dimensions. Conventional miniaturization techniques like half‐mode substrate‐integrated waveguides [43] achieve 50% size reduction but incur open‐boundary radiation losses. All these limitations have prevented the realization of a unified platform that simultaneously achieves topological protection, seamless waveguide interfacing, and compactness.

Gap waveguide (GW) technology [44, 45, 46, 47, 48] provides non‐contact electromagnetic (EM) wave confinement through periodic soft walls. It eliminates conductive losses while simplifying fabrication. Building upon the structural similarities between GWs and VPCs, we develop an ultracompact valley‐ridge gap waveguide (VRGW) that supports a topological half‐supermode through the integration of valley Hall state with ridge waveguide mode. This innovative VRGW platform simultaneously resolves two longstanding challenges in integrated topological waveguides: direct mode matching between topological edge states and conventional TE_10_ modes, as well as device miniaturization without performance degradation. To achieve this, we first establish an odd‐symmetric topological supermode through controlled valley‐ridge coupling. By implementing a perfect electric conductor (PEC) in the symmetry plane, a leakage‐free 50% size reduction is achieved while preserving mode integrity. Next, optimized VPC structures along the ridge facilitate direct mode matching between topological half‐supermodes and conventional TE_10_ modes within the whole bandgap. Experiment results verify robust operation with reflection losses below −15 dB. The proposed platform exhibits exceptional compactness, supporting sharp‐bend signal routing without degradation. These results represent the first practical implementation of topological half‐supermodes in integrated waveguide. Our work establishes a new platform for high‐performance wireless communications that overcomes the limitations of topological waveguides in size, bandwidth, and fabrication complexity.

## 2 Results

### 2.1 The VRGW

**FIGURE 1:**
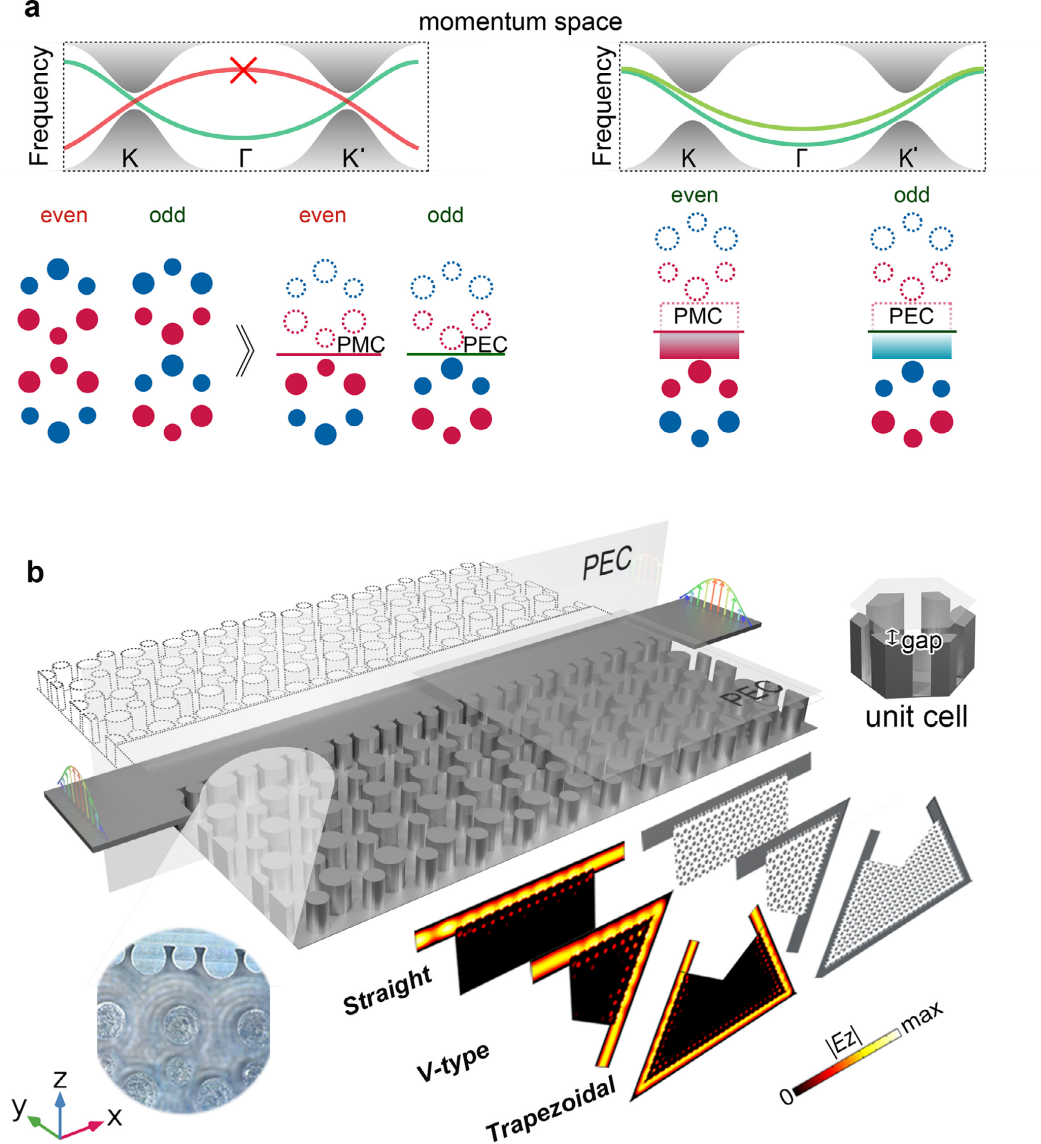
Design and operational principles of the VRGW. (a) Formation mechanism of topological half‐supermodes. Left: Original valley kink states exhibit symmetry‐dependent properties (even‐symmetry with concave dispersion curve, truncatable by a PMC boundary and odd‐symmetry with convex dispersion curve, truncatable by a PEC boundary). Right: Hybrid supermodes emerge from the coupling between odd‐symmetry valley kink states and ridge waveguide modes, maintaining convex dispersion curves while remaining separable via PEC/PMC boundaries. (b) Implemented VRGW prototype enabling direct TE_10_ mode waveport integration and supporting three distinct unidirectional propagation paths (straight, V‐type, and trapezoidal). Inset: (Left) High‐resolution image of the fabricated metallic structure showing integrated VPC and ridge patterns achieved through CNC milling; (Right) VPC unit cell constructed using GW technology.

Valley kink states exist at the interface of two VPCs with distinct topologies, where the group velocity of the kink states around each valley is governed by the valley Chern number difference. As shown in Figure 1a, within the nontrivial bandgap, valley kink states exhibiting a convex shape display odd‐symmetry, whereas those with a concave shape adopt even‐symmetry. In this work, we find that ridge waveguide modes only couple with valley kink states with a convex shape, establishing hybrid even‐ and odd‐symmetry supermodes (both maintaining convex shape) that can be isolated using symmetry‐matched boundaries (PEC for odd modes, perfect magnetic conductor (PMC) for even modes). The proposed system is implemented using GW technology in Figure 1b, operating in the frequency range of 24.5–27 GHz. To enable compact operation and suppress leakage, a PEC boundary condition is employed. The proposed VRGW achieves 50% transverse size reduction compared to conventional and topological waveguides [37, 40, 41, 42, 48] while maintaining robust topological protection for signal transmission through sharp bends. Furthermore, by engineering the ridge‐VPC interface, direct TE_10_ mode matching is achieved without requiring transition structures, facilitating seamless integration with existing waveguide systems. The VRGW is fabricated as a monolithic metallic structure through computer numerical control (CNC) milling, integrating VPC and ridge patterns as illustrated in the left inset of Figure 1b.

### 2.2 Valley Phase Transition in VPCs

**FIGURE 2:**
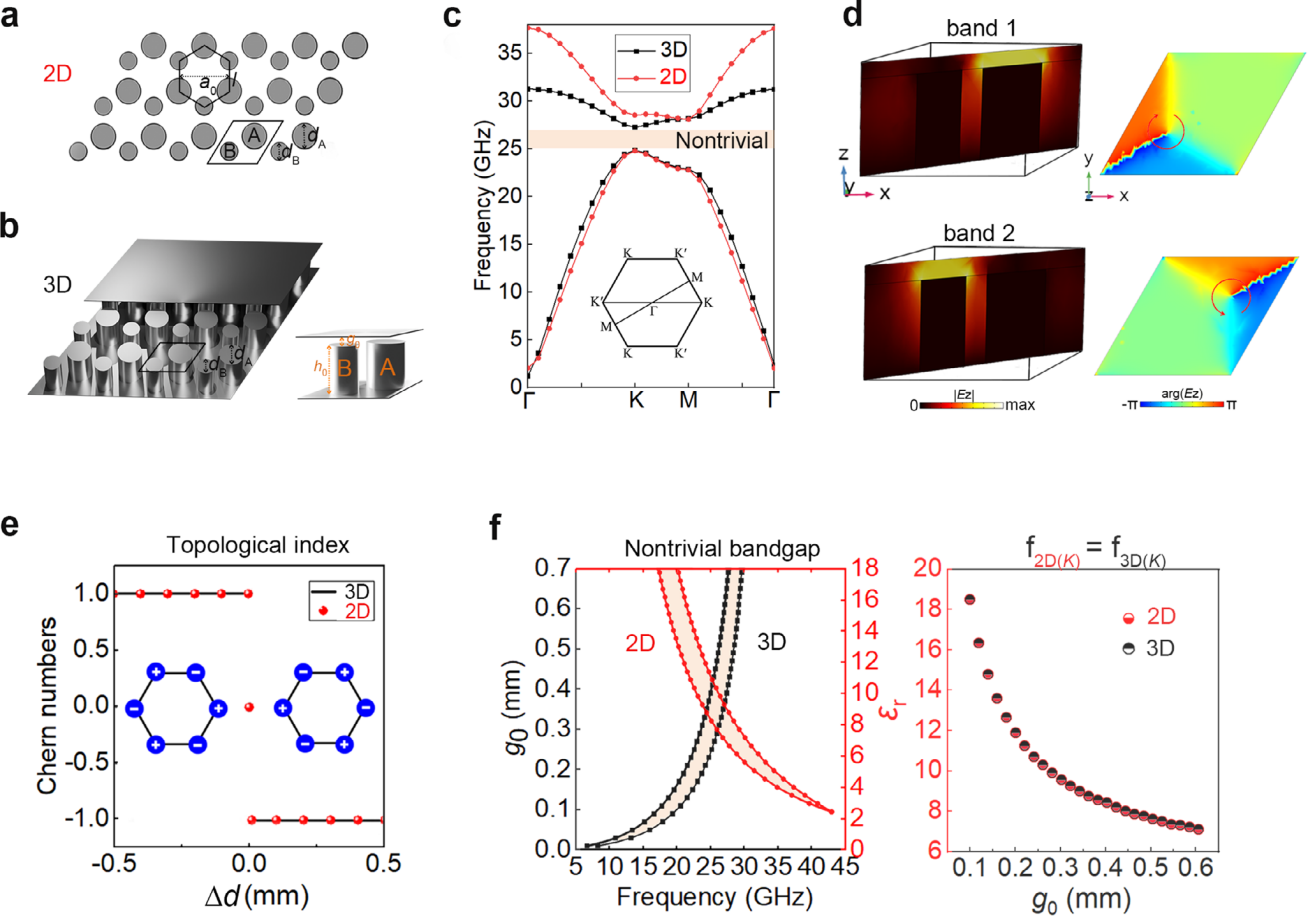
Equivalence between the 2D dielectric VPC and the 3D metallic VPC. (a) 2D dielectric VPC. It is composed of two types of rods with diameters of *d*_A_ and *d*_B_. (b) 3D metallic VPC. It is composed of two types of finite‐height pins with diameters of *d*_A_ and *d*_B_. The pins are sandwiched between two metallic plates with the bottom one connected and the top one detached. The *h*_0_ and *g*_0_ are the height of the pins and gap, respectively. (c) Band structures of 2D dielectric VPC (red line) and 3D metallic VPC (black line). (d) The amplitude and phase distributions of *E*_z_ of the 3D metallic VPC at *K‐*valley of bands 1 and 2. (e) The variations of the valley Chern number with respect to the difference (Δ*d*  =  *d*_A_ −  *d*_B_) between *d*_A_ and *d*_B_. The lines and dots represent the results from the 3D metallic VPC and 2D dielectric VPC, respectively. (f) The variations of the bandgap with respect to key parameters of the 2D dielectric VPC (*ε*_r_) and 3D metallic VPC (*g*_0_) and their mappings. Parameters: *a*_0_ = 3.5 mm, *l* = $a_{0}/\sqrt{3}$, *d*_A_= 1.7 mm, *d*_B_ = 1.2 mm, *ε*_r_ = 8.5, *h*_0_ = 1.85 mm and *g*_0_ = 0.38 mm.

To start with, a 2D VPC is mimicked using the 3D GW structures. This platform confines the EM field to a quasi‐2D plane, thereby successfully hosting the desired topological valley edge states. Figure 2a,b shows the geometries of the 2D dielectric VPC and its 3D metallic VPC counterpart. The 2D dielectric VPC is arranged in a triangular lattice (lattice constant *a*_0_ = 3.5 mm) with a unit cell (black rhombus) containing two dielectric rods (A and B) of relative permittivity *ε*_r_ = 8.5 and diameters *d*_A_ and *d*_B_. The 3D metallic VPC maintains the same lattice arrangement but consists of metallic pins sandwiched between two parallel metallic plates. These metallic pins preserve the same diameter parameters as those of the rods in the 2D VPC but have a finite height *h*_0_ = 1.85 mm. Notably, the pins are connected to the bottom plate while remaining separated from the top plate by a controlled gap (*g*_0_ = 0.38 mm).

To modulate the valley degree of freedom, the inversion symmetry of both VPCs is broken by varying the diameters of the rods or pins. As shown in Figure 2c, both VPCs exhibit a common bandgap between 25 and 27 GHz when *d*_A_ = 1.7 mm, *d*_B_ = 1.2 mm. Eigenmodes of band 1 and band 2 at the *K*‐valley for the 3D VPC are shown in Figure 2d. To ensure a valid quasi‐2D approximation, the gap size *g*_0_ must be kept below a cutoff that suppresses the propagation of all parallel‐plate modes, not merely the fundamental TEM mode. With a properly selected *g*_0_, the electric‐field (*E*_z_) magnitude exhibits a quasi‐2D distribution (Figures S1 and S2). Simultaneously, its phase displays a distinct vortex‐like profile. This behavior indicates the existence of a non‐zero valley Chern number. These topological characteristics show excellent agreement with those of the 2D VPC. Figure 2e presents the valley Chern number *C*_V_ as a function of the rod or pin diameter difference (Δ*d* = *d*_A_ − *d*_B_). The topological indices *C*_K, K′_ = ±1/2 shown in the insets (Figure S3) yield valley Chern numbers *C*_V_ = (*C*_K_ − *C*_K′_) = ±1, demonstrating identical topological phase transitions in both structures. This result conclusively establishes their equivalent bulk topological properties. The topological band boundaries of the 3D VPC (black squares, Figure 2f) can be modulated by varying the gap size *g*_0_, which is analogous to the 2D VPC (red points) when its permittivity *ε*_r_ is tuned. When synchronous tuning is satisfied (*f*_2D _ = *f*_3D_ at the *K*‐valley of band 1), a direct correlation emerges between *g*_0_ and *ε*_r_. This *g*_0_ − *ε*_r_ mapping enables controlled transformation of material properties while maintaining intrinsic topological phase characteristics of the system.

### 2.3 Half‐Supermodes in VRGW

Conventional waveguides typically support fundamental TE_10_ modes. Structural miniaturization can be achieved by bisecting the waveguide along its center propagation plane, where the resulting open boundary effectively mimics a PMC condition [43]. However, this approach inevitably suffers from radiation losses, especially at bends. In contrast, VPCs naturally support odd‐symmetric topological modes that are compatible with PEC boundary conditions. While this suggests a more robust path to miniaturization, practical implementation has remained elusive due to persistent challenges in mode matching and device fabrication. We address these challenges by realizing a half‐supermode through engineered coupling between ridge waveguide modes and valley kink states.

**FIGURE 3:**
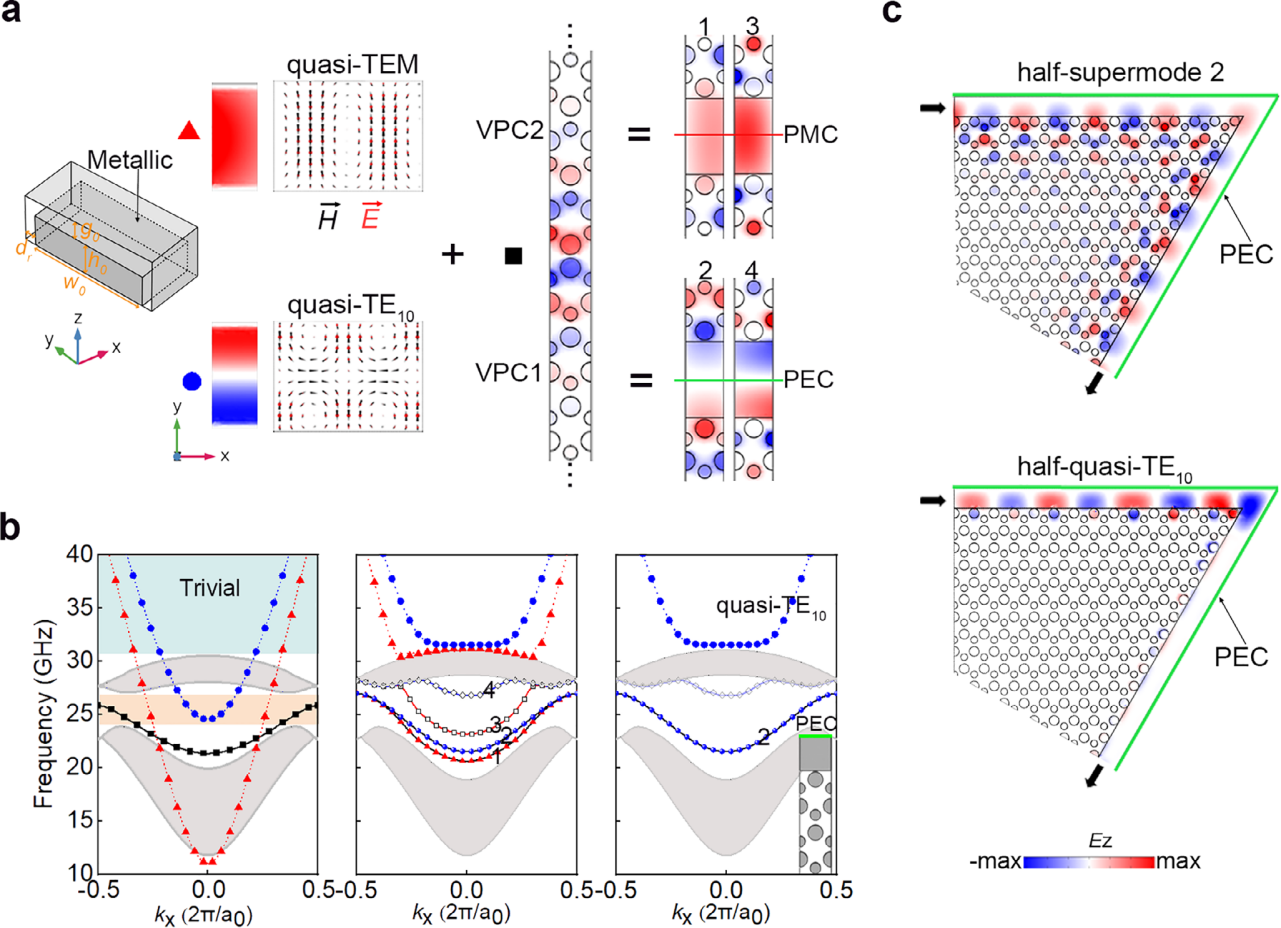
Formation and transmission of topological hybrid half‐supermodes. (a) Mode formation process: (Left) Ridge waveguide supporting fundamental quasi‐TEM and quasi‐TE_10_ modes, with arrows indicating the distributions of $\underset{E}{\to }$ (red) and $\underset{H}{\to }$(black) vectors; (Middle) VPC1|VPC2 interface with odd‐symmetry valley kink state; (Right) Hybridized supermodes resulting from coupling between the ridge and VPC1|VPC2 interface. (b) Band structure evolution: (Left) Individual ridge waveguide and VPC1|VPC2 interface; (Middle) Coupled valley‐ridge system; (Right) PEC‐truncated valley‐ridge system. (c) Comparison of *E_z_* transmission through V‐type waveguide paths: (Top) Topological half‐supermode 2 showing robust propagation; (bottom) Conventional half‐quasi‐TE_10_ mode showing bend‐induced losses. Parameters: *h*_0_ = 1.85 mm, *w*_0_ = 3.5*l*, *g*_0_= 0.38 mm and *d*_r_ = 0.625 mm. Transition between VPC1|VPC2 domain wall configurations is achieved by adjusting the equivalent ridge‐sidewall distance *d*_r_ = 0.625 mm (Figure S4).

Using identical pin height and gap dimensions from the 3D VPC unit cell in Figure 2b, a conventional ridge waveguide is designed first to support two modes (left panel, Figure 3a). Field vector analysis reveals that the even‐symmetric fundamental mode exhibits quasi‐TEM characteristics, while the odd‐symmetric first higher‐order mode shows quasi‐TE_10_ behaviour (Figure S4). Both modes operate within the target bandgap (red and blue curves, left panel, Figure 3b). The VPC1 (Δ*d* = 0.5 mm)|VPC2 (Δ*d* = −0.5 mm) interface is designed from the 3D VPC unit cell (middle panel, Figure 3a). The shared convex dispersion profiles of ridge modes and valley states enable mode coupling in the nontrivial bandgap. Integrating the ridge waveguide with the VPC1|VPC2 interface forms the complete valley‐ridge structure, yielding four hybrid supermodes through interaction between the two ridge mode bands and a single valley kink state band (right panel, Figure 3a). These supermodes are numerically labeled from 1 to 4 according to their increasing eigenfrequencies at *k_x_* = 0 (middle panel, Figure 3b).

The system produces two pairs of characteristic supermodes. The first pair, modes 1 and 3, originates from coupling between the valley kink state and the quasi‐TEM ridge mode while preserving the even‐symmetry of the original quasi‐TEM mode. It can be observed that mode 1 localizes primarily in the ridge and larger interface pins, whereas mode 3 concentrates in the ridge and smaller interface pins. This behavior is consistent with the perturbation theory. The second pair, modes 2 and 4, results from coupling between the valley kink state and quasi‐TE_10_ ridge mode. These supermodes maintain the odd‐symmetry of quasi‐TE_10_ modes. They exhibit spatial distributions similar to modes 1 and 3, but with their respective symmetry properties.

Notably, besides the nontrivial bandgap containing hybridized supermodes, there is a higher‐frequency trivial bandgap that overlaps with the mode frequencies of the ridge waveguide. Within this trivial bandgap region, the fundamental quasi‐TEM and quasi‐TE_10_ modes of the ridge waveguide maintain their original form without hybridization with VPC states. This preservation stems from the absence of valley kink states in the trivial bandgap, leaving the ridge mode characteristics undisturbed. Interestingly, the ridge waveguide modes cannot couple with the convex valley kink states at the VPC2|VPC1 interface due to topological constraints. As a result, the VPC2|VPC1 configuration effectively acts as a conventional periodic cladding, creating only a trivial stopband for the ridge GW (Figure S7).

The even‐ and odd‐symmetry supermodes are isolated by introducing PEC or PMC boundaries along the central plane. As shown in the right panel of Figure 3b, the odd‐symmetry supermodes (modes 2, 4, and quasi‐TE_10_) are selectively preserved when applying a PEC boundary, while the even‐symmetry supermodes (modes 1, 3, and quasi‐TEM) require a PMC boundary (Figure S5). Full‐wave simulations verify the robustness of the supermode by comparing transmission in nontrivial versus trivial bandgaps. At 120° V‐bends, the half‐supermode (Mode 2) maintains excellent stability and high transmission efficiency (top panel, Figure 3c). In contrast, the conventional half‐quasi‐TE_10_ mode in the trivial bandgap suffers significant bending loss (bottom panel, Figure 3c). These results demonstrate the superior bend immunity of topologically protected supermodes for robust waveguide applications.

### 2.4 Mode Matching and Experimental Validation

**FIGURE 4:**
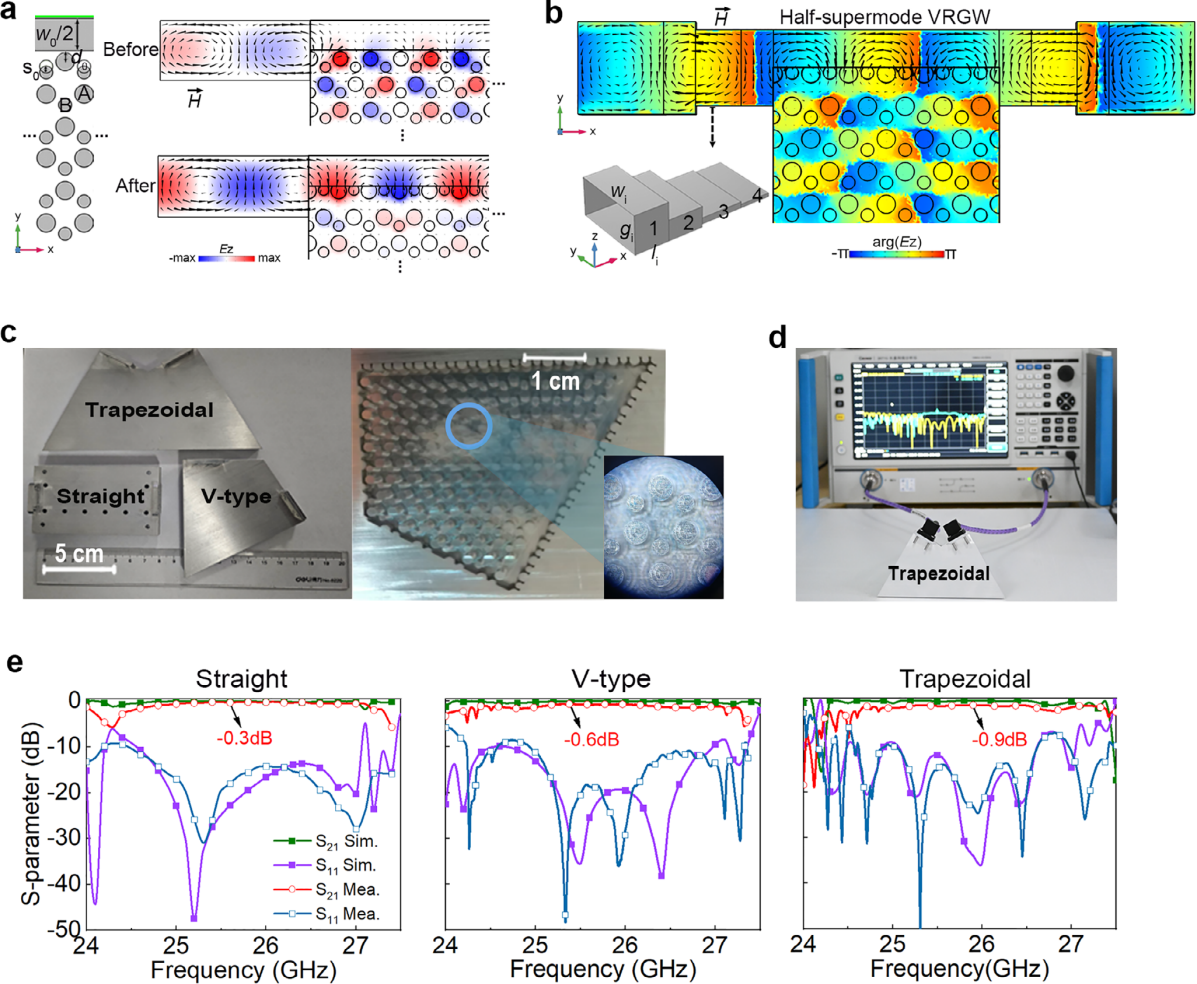
Full‐wave simulations and experimental characterizations of half‐supermodes in VRGWs. (a) Matching between rectangular waveport and VRGW before and after optimization. *E*_z_ and $\underset{H}{\to }$(black arrows) are shown. *w*_0_/2 signifies the width of the ridge, *d*_0_ represents the distance from the ridge‐sidewall to the A pin's center, and *s*_0_ indicates the displacement of the B pins. The parameters of the final VRGW: *w*_0_ = 3.5*l*, *d*_0_= (*l* − *d*_A_)/2, *s*_0_ = 1.1 mm. The waveport has an aperture size of 7.1 × 0.38 mm^2^. (b) Simulated *E*_z_ phase and $\underset{H}{\to }$ lines in the straight VRGW connected with standard waveguides. The inset is a 3D view of the stepped transition waveguide connecting the VRGW to WR34 (BJ260). (c,d) Photographs of the three fabricated VRGW prototypes, highlighting their internal and external details. Scale bars: 5 cm (Left), 1 cm (Right). The S‐parameters were measured using a Caeyear‐3671G VNA with a WR34 (BJ260) to 2.4 mm female connector adapter. (e) The simulated and measured S‐parameters of all three VRGWs.

To enable direct TE_10_‐mode excitation, we implement a waveport in direct contact with the VRGW, matching its height to the gap dimensions of VRGW while maintaining a shared PEC boundary condition. The valley–ridge interface provides critical additional degrees of freedom for mode matching. As shown in Figure 4a, before tuning, there is a severe mode mismatch between the TE_10_‐mode and half‐supermode 2. The *E*_z_ amplitudes and $\underset{H}{\to }$(black arrows) exhibit significant divergence. Through precise optimization of the positions of edge pins, simultaneous *E*_z_ and $\underset{H}{\to }$ alignment with the TE_10_‐mode is achieved. Importantly, as shown in Figure 4b, the chiral phase nature of VPCs is retained, confirming preservation of bulk topological properties. This confirms robust topological protection throughout the mode‐matching process, enabling efficient excitation without compromising valley‐dependent properties.

For experimental verification, we implemented a test platform incorporating standard WR34 (BJ260) waveguides with an aperture size of 8.64 × 4.32 mm^2^. To achieve optimal impedance matching with our custom rectangular waveguide (7.1 × 0.38 mm^2^ aperture), we developed a stepped transition waveguide (inset of Figure 4b; Figure S8). Full‐wave simulations demonstrate efficient power transmission. Figure 4b shows stable mode transmission through the smooth evolution of magnetic field patterns at the interface. Three VRGW prototypes were fabricated for experimental validation, namely straight, V‐type (120° bend), and trapezoidal (two 120° bends) configurations. The straight waveguide achieves 50% lateral size reduction compared to conventional designs while maintaining complete mode confinement without radiation loss. The bent configurations exhibit even greater compactness (>50% size reduction) while preserving performance. Their transmission efficiency was characterized using a vector network analyzer (VNA) as depicted in Figure 4c,d. Experimental results in Figure 4e indicate that insertion losses are consistently maintained at 0.3 dB for all three configurations. This remarkable bending immunity stems from valley vortex phase protection, which effectively suppresses backscattering. Compared to conventional waveguides (quasi‐TE_10_) performance (Figure S9), our design demonstrates superior robustness for power transmission in complex integrated circuit geometries.

### 2.5 Verifications on Telecommunication System

**FIGURE 5:**
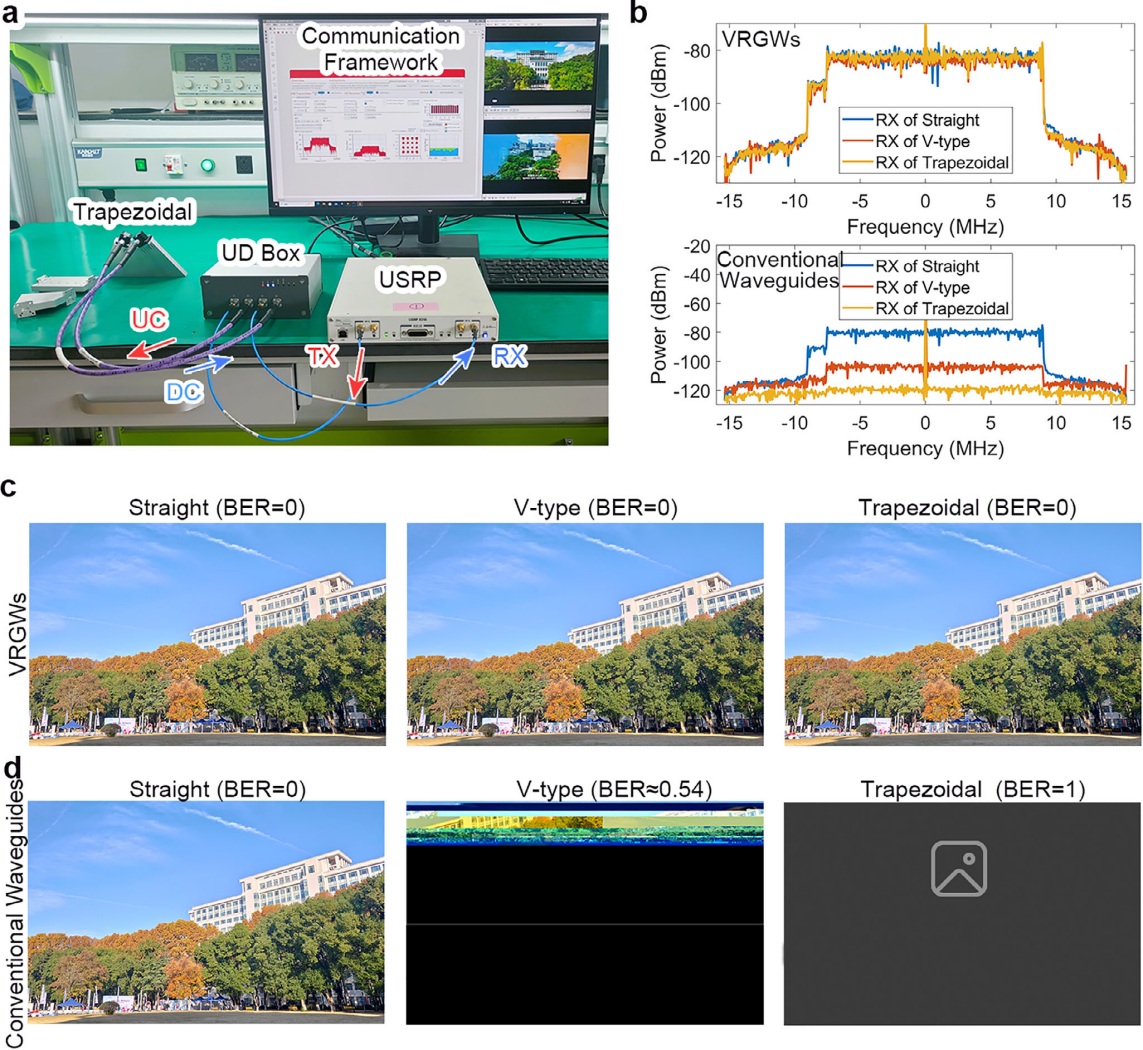
Telecommunication experiments. (a) The experimental platform of telecommunication system. (b) The Rx power spectrum of VRGWs (upper panel) and conventional waveguides (lower panel). (c, d) The received images and BERs through three VRGWs and corresponding conventional waveguides.

To visually validate the bending‐immune transmission characteristics of the proposed VRGWs, a telecommunication system was established to compare their performance with conventional waveguides in image transmission. Figure 5a illustrates the experimental telecommunication system, which consists of a universal software radio peripheral (USRP), a TMYTEK UD Box frequency multiplier, and a telecommunication framework on a general‐purpose computer. The computer transmits (Tx) data to the USRP via a PCI Express interface. The baseband signal is encoded using 64‐QAM modulation in the USRP, followed by carrier up‐conversion (UC) to work frequency through the UD Box frequency multiplier. The up conversion and down conversion operations of the UD box only alter the carrier frequency without modifying the modulation scheme, encoding, and bandwidth of the signal. The three VRGWs and conventional waveguides (straight, V‐type, and trapezoidal) are sequentially connected in telecommunication system for measurement. The received signals undergo down‐conversion (DC) via the UD Box frequency multiplier and are sent back to the USRP for demodulation.

The up‐envelopes of received (Rx) power signals are depicted in Figure 5b (for original signal power of USRP ports, see Figure S10). The upper panel illustrates the Rx power of VRGWs, which are almost identical, indicating that the VRGWs transmit signals robustly. The lower panel illustrates the Rx power of conventional waveguides (quasi‐TE_10_). While the straight waveguide transmits signals normally, the V‐type performs poorly, and the trapezoidal one completely loses the signal. Spectrum analysis (Figure 5b) reveals that the Rx power of conventional waveguides exhibits significant attenuation (−80–−120 dBm) within the 20 MHz bandwidth as bends are introduced. In contrast, the VRGWs maintain stable power efficiency at −80 dBm due to their topological protection mechanism, conclusively demonstrating their transmission robustness in complex pathways. Figure 5c,d illustrates the experimental results of received images using the telecommunication system. The images received are without distortions for the three VRGWs, while those of the conventional waveguides become worse when bends are introduced, e.g., most of the information is lost in V‐type, and the image is completely unrecognizable in the trapezoidal. The bit error rates (BER) for these three transmission scenarios were 0, 0.54188 (the image has a total of 132920064 bits with 72027353 error bits.) and 1, respectively, confirming that conventional waveguide performance decreased progressively as the bends increased. Additionally, real‐time video transmission capability is experimentally validated, with a demonstration provided in Video S1 (Telecommunication_experiment.mp4). The topological nature of half‐supermodes provides robust guided‐wave transmission, enabling propagation through disordered geometries and structural imperfections while maintaining signal integrity. This robustness substantially improves system reliability and enables high‐performance communication and signal‐processing applications.

## 3 Conclusion

In conclusion, we have theoretically proposed and experimentally demonstrated a VRGW that fundamentally addresses two critical challenges in merging topological photonics with conventional waveguide systems. First, we resolve the inherent symmetry mismatch between topological edge states and conventional waveguide modes by engineering topological half‐supermodes through controlled coupling of ridge‐waveguide modes with valley kink states. Second, we eliminate excessive device dimensions via a PEC‐truncated design, achieving 50% size reduction while preserving full mode confinement. All these features are essential for integrated waveguide systems. Measurements demonstrate direct TE_10_‐mode matching and robust backscattering suppression in three typical VRGW samples. This work successfully bridges the gap between topological photonics and waveguide engineering, enabling compact, high‐performance telecommunication systems with inherent immunity to disorder and bends.

## 4 Methods

### 4.1 Sample Fabrication

The samples are composed of two polished aluminum plates. The bottom plate is precision‐milled using a CNC machine to create a 3D VPC with specific lattice parameters, featuring dimensions of *d*_A_ = 1.7 mm, *d*_B_ = 1.2 mm, *a*_0_ = 3.5 mm, and *h*_0_ = 1.85 mm. The trapezoidal transition sections at both ends are also crafted on the bottom plate. The top plate is milled to have a groove structure with a height of *g*_0_ = 0.38 mm.

### 4.2 Numerical Calculation

The dispersion band calculation, eigenmode distribution, and EM field distribution shown in this work are calculated by a 3D finite element method using the optical module of commercial software COMSOL MULTIPHYSICS.

### 4.3 Experimental Apparatus

We employed a Vector Network Analyzer (VNA) [Ceyear‐3671G] to measure the S‐parameters of the waveguide. During the experiment, we initially calibrated the VNA using two 2.4 mm connecting cables to ensure the accuracy of the measurement results. Subsequently, two standard coaxial‐to‐waveguide adapters WR34 (BJ260) were mated and calibrated again to eliminate any transmission losses introduced by the WR34. After this step, the test waveguide was connected to the ports of the WR34, and S‐parameter measurements were conducted over the specified frequency range. Throughout the measurement process, the VNA provided detailed information regarding the waveguide's reflection coefficient, transmission coefficient, and impedance characteristics. The software and hardware in telecommunication experiments are the National Instruments (NI) LTE Application framework, NI x310 USRP, and TMYTEK UD Box frequency multiplier.

## Author Contributions

All authors contributed extensively to the work presented in this paper. H. L. and M.L.N.C. supervised all aspects of this work. R.Z. and M.L.N.C. managed this project. R.Z. carried out the theoretical analysis and experiments with the assistance of X.T.S., M.L.N.C., and Z.H.L. And H.L., Y.R., Z.H.Y, and J.J. put inputs together from all other coauthors in the paper revision.

## Conflicts of Interest

The authors declare no conflicts of interest.

## Supporting information




**Supporting File**: advs73498‐sup‐0001‐SuppMat.docx.

**Supporting File**: advs73498‐sup‐0002‐Telecommunication Experiment_lsp.mp4.

## Data Availability

The data that support the findings of this study are available on request from the corresponding author. The data are not publicly available due to privacy or ethical restrictions.
