# Supplementary material for: Super‐Robust Telecommunications Enabled by Topological Half‐Supermodes
Source: Adv Sci (Weinh). 2026 Jan 9;13(13):e15157. doi: 10.1002/advs.202515157 (PMC12955864; doi:10.1002/advs.202515157)
Supplement: Supplementary file 1 — Supporting File: advs73498‐sup‐0001‐SuppMat.docx. [file ADVS-13-e15157-s001.docx]

Supporting Information

Super-robust telecommunications enabled by topological half-supermodes

Rui Zhou†, Xintong Shi†, Hai Lin*, Yan Ren, Hang Liu, Zihao Yu, Jing Jin, Zhihao Lan and Menglin L. N. Chen*†

**Table of contents**

[**S1. Field Distribution in 3D Metallic VPC. 20**](#_Toc215509907)

[**S2. Numerical calculation of Berry curvature. 22**](#_Toc215509908)

[**S3. Equivalent ridge waveguide (RW) in the VRGW 23**](#_Toc215509909)

[**S4. Half-supermodes with PMC/PEC boundary 24**](#_Toc215509910)

[**S5. VPC1|VPC2 interface combined ridge waveguide 25**](#_Toc215509911)

[**S6. VPC2|VPC1 interface combined ridge waveguide (RW) 26**](#_Toc215509912)

[**S7. Stepped transition rectangular waveguide 27**](#_Toc215509913)

[**S8. Transmission characteristics of conventional waveguide 28**](#_Toc215509914)

[**S9. Original signal power of USRP ports 29**](#_Toc215509915)

[**References 30**](#_Toc215509916)

# **S1. Field Distribution in 3D Metallic VPC.**


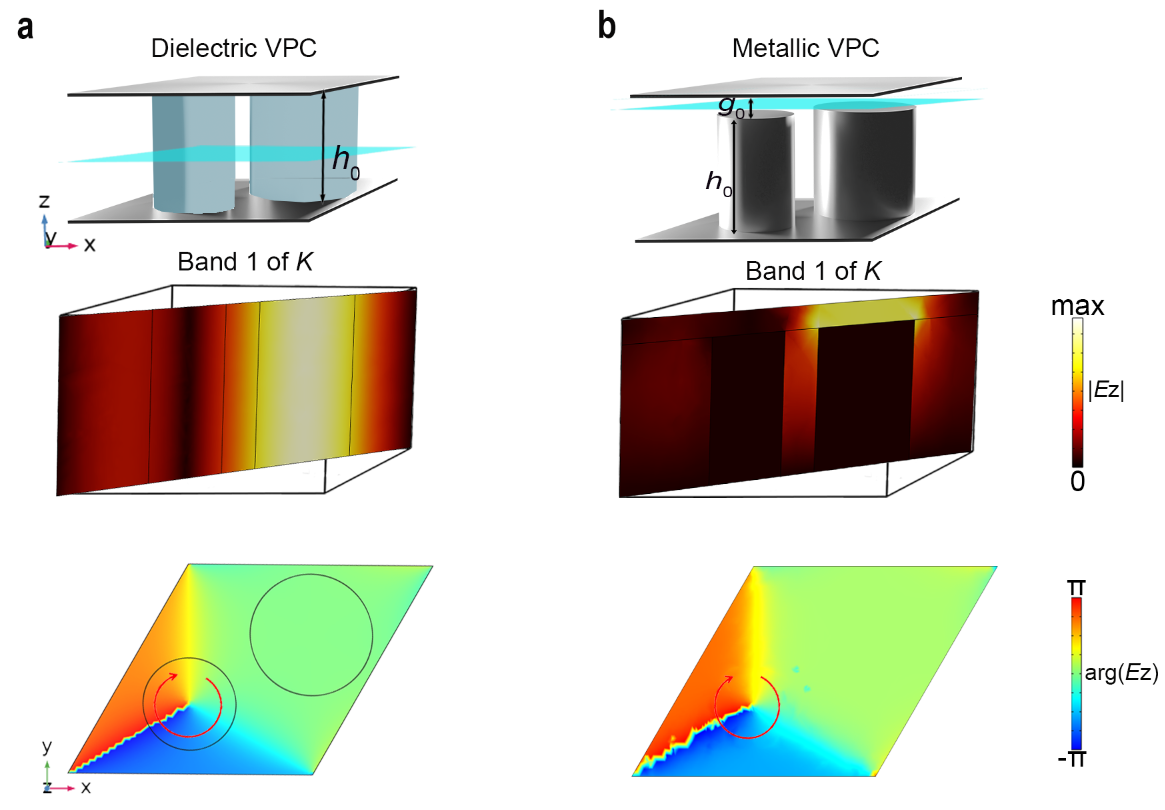


**Figure S1**ⅠThe electric-field amplitude and phase distributions of the dielectric VPC (a) and metallic VPC (b) at the *K*-valley of band 1.

To visually demonstrate the electric-field (*E*z) magnitude exhibits a quasi-2D distribution within the gap region of the metallic VPC, we constructed a corresponding dielectric VPC structure (with a height of *h*_0_ = 1.524 mm) sandwiched between metal plates. **Figure S1** shows the *E*z and phase distributions of both structures ($\Delta d$= 0.5 mm) at the *K*-valley of band 1. In the dielectric VPC (Fig. S1(a)), the *E*z is uniformly confined inside the dielectric rods and remains consistent along the z-axis, exhibiting typical 2D confinement characteristics and a distinct vortex phase. In the metallic VPC (Fig. S1(b)), the *E*z is primarily concentrated in the gap region but displays a highly similar spatial distribution pattern and a complete vortex phase.

These results indicate that the *E*z localized in the gap of the metallic VPC is equivalent in behavior to that in the dielectric VPC, thereby confirming the quasi-2D distribution of the *E*z in the metal structure.


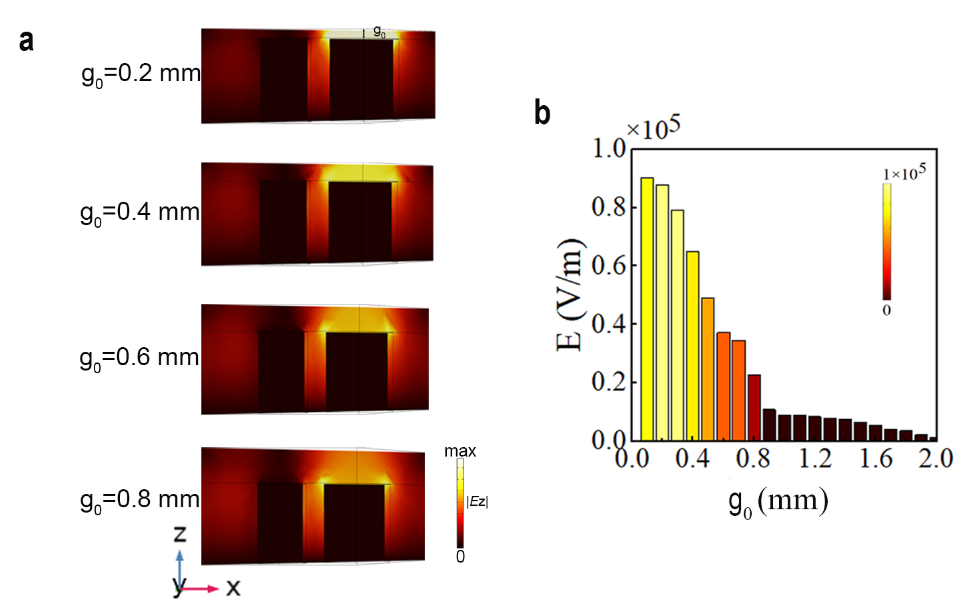


**Figure S2**ⅠEvolution of the uniformity (a) and amplitude (b) of the *E*z field in the 3D VPC with increasing gap height *g*_0_.

The distribution of the quasi-2D *E*_Z_ are primarily governed by the gap height *g*_0_. As shown in **Figure S2**, as *g*_0_ increases, the *E*_Z_ uniformity gradually decreases. Quantitative analysis further reveals that when *g*_0_ reaches 0.6 mm, the *E*_Z_ amplitude decays by approximately 50%. To ensure optimal equivalence performance while accounting for fabrication tolerances, a value of *g*_0_ =0.38 mm was selected as the optimized parameter in this manuscript.

# **S2. Numerical calculation of Berry curvature.**


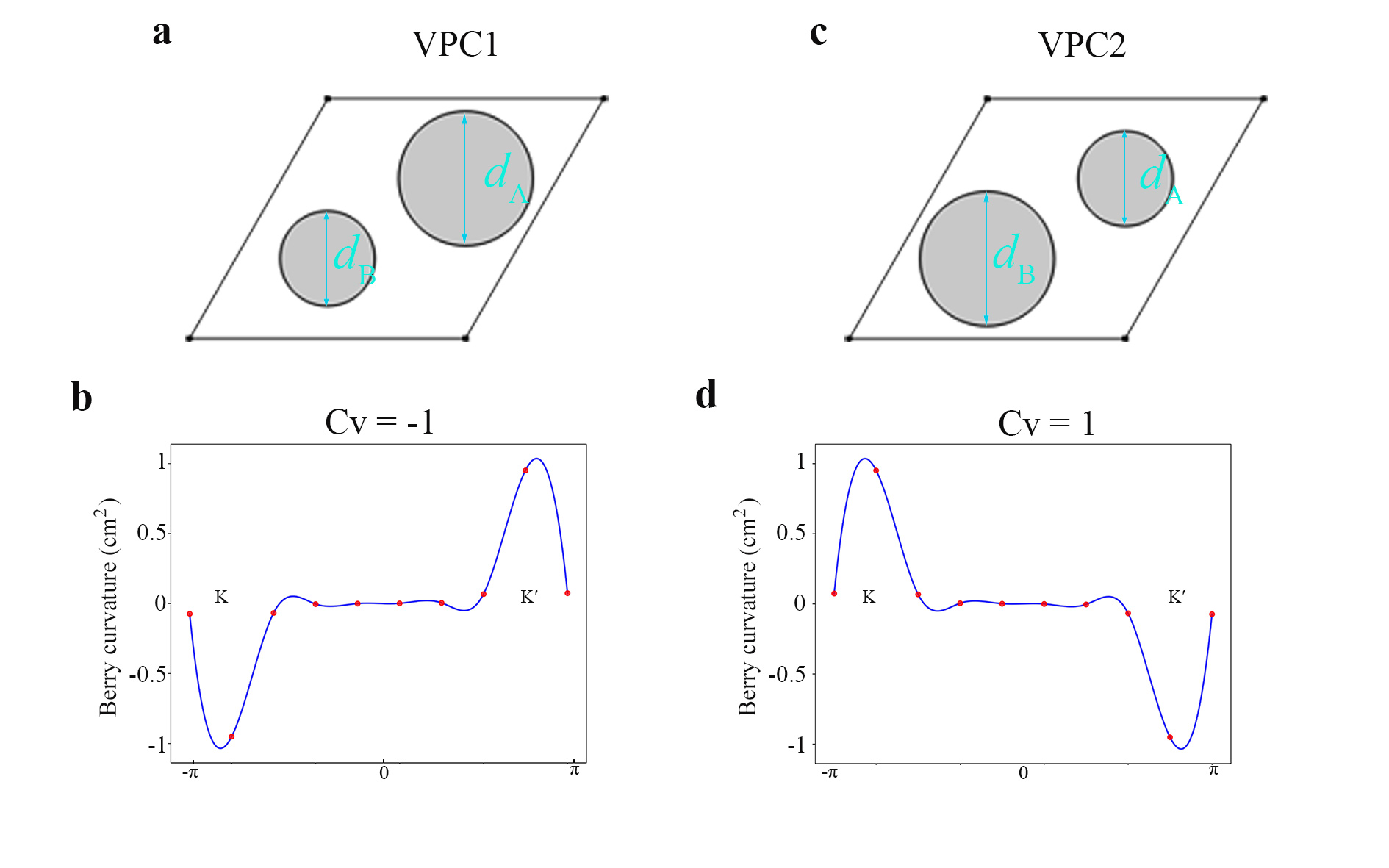


**Figure S3**Ⅰ**Berry curvature distribution of the VPCs.** (a) The unit cell of VPC1, where *d*_A_=1.7 mm, *d*_B_=1.2 mm. (b) The distribution of the Berry curvature of VPC1 near the *K/K*' valley in the FBZ. (c) The unit cell of VPC2, where *d*_A_=1.2 mm, *d*_B_=1.7 mm. (d) The distribution of the Berry curvature of VPC2 near the *K/K*' valley in the FBZ.

We will classify the topological states of VPCs with inversion asymmetry by computing their non-zero Berry curvature and valley Chern number from first principles. The Berry connection of the lowest band can be defined as:^[S1]^

$\vec{A}(\vec{k})\equiv i\left\langle u_{\vec{k}} \right|\nabla_{\vec{k}}\left| u_{\vec{k}} \right\rangle=i\text{∮}_{\text{unitcell }}d^{2}r\varepsilon(\vec{r})u_{\vec{k}}^{*}\left[ \nabla_{\vec{k}}u_{\vec{k}} \right]$………………………(E1)

where $u_{\vec{k}}$ is the electromagnetic fields, an asterisk denotes complex conjugation, and $\varepsilon\left( \vec{r} \right)$is the spatial permittivity distribution. Note that the eigenstate amplitude is normalized such that $\left\langle u_{\vec{k}_{k}}\mid u_{\vec{k}} \right\rangle=1$ and that phases of each eigenstate should be normalized by keeping the phase constant at one arbitrary point. The gauge-independent Berry curvature $\Omega(\vec{k})$ is obtained by:

$\Omega(\vec{k})\equiv\nabla_{\vec{k}}\times\vec{A}(\vec{k})=\frac{\partial A_{y}(\vec{k})}{\partial k_{x}}-\frac{\partial A_{x}(\vec{k})}{\partial k_{y}}$…………………..…………(E2)

**Figure S3** shows that the calculated Berry curvature near the *K* valley of the lowest band, is opposite to that near the *K*′ valley. Topological indices *C*_K_/_K_′ at the *K* and *K*′ valleys are defined as the integration of the Berry curvature within a half first Brillouin zone (FBZ), and the valley Chern number is defined as *C*_V_ = (*C*_K_ - *C_K_*_′_). As a result, the valley-Chern number *C*_V_ = (-1/2) - (1/2) = -1 for VPC1, and *C*_V_ = 1 for VPC2.

# **S3.** **Equivalent ridge waveguide (RW) in the** **VRGW**


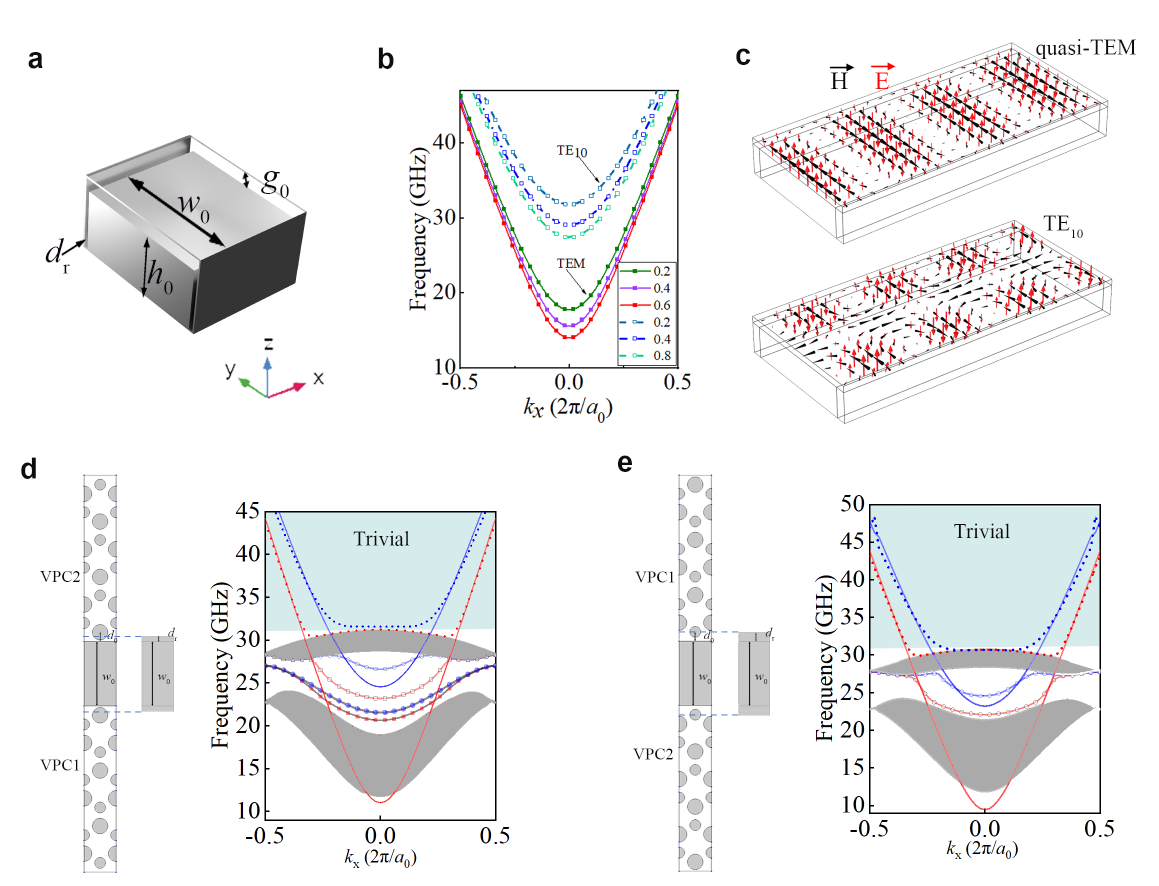


**Figure S4**Ⅰ**Ridge mode distributions.** (a) 3D structure diagram of a RW. (b) Band structure of the quasi-TEM (solid curve) and quasi-TE_10_ (dashed curve) modes varies with changes in ridge-sidewall distance (*d*_r_). (c) The quasi-TEM and quasi-TE_10_ electric ($\vec{E}$) and magnetic ($\vec{H}$) field vectors distributions. (d, e) Schematic and dispersion bands of combined waveguide (dashed curve) and equivalent RWs (solid curve) in the trivial bandgap. The combined waveguide with different VPC2|VPC1(in (d)) or VPC2|VPC1(in (e)) domain walls. Parameters: *h*_0_ =1.85 mm, *w*_0_ = 3.5*l*, *g*_0_= 0.38 mm, *d*_0_ = (*l*-*d*_A/B_)/2 and *d*_r_ = 0.9 mm or 0.625 mm.

3D view of the ridge waveguide(RW) supercell is shown in **Figure S4(a)**. As *d*_r (_ridge-sidewall distance) gradually decreases, the fundamental mode (solid curve) and first higher-order mode (dashed curve) cutoff frequencies will shift upward, as shown in **Figure S4(b)**. Observations reveal that the $\vec{E}$ of the fundamental mode is zero in the propagation direction *x*. Similarly, the $\vec{H}$ is zero on the *x*-axis. Moreover, the $\vec{E}$ distribution is perpendicular to the $\vec{H}$, and the fundamental mode is identified as a quasi-TEM mode, as shown in **Figure S4(c)**. Using the same method, it can be determined that the first higher-order mode is an odd-symmetric TE_10_ mode.

Study^[S2]^ indicates that the dispersion distribution of conventional ridge gap waveguide can be approximated by equivalent RW dispersion. When the RW dispersion band (*w*_0_ = 3.5*l*, solid curve) overlaps with the VRGW dispersion (*w*_0_ = 3.5*l*, *d*_0_ = *l/*2, dashed curve) in the trivial bandgap, it serves as the equivalent case, as shown in **Figures S3(d, e)**. **Figure S3(d)** corresponds to an equivalent *d*_r_ = 0.625 mm, while **Figure S4(e)** corresponds to an equivalent *d*_r_ = 0.9 mm.

#

# **S4. Half-supermodes with PMC/PEC boundary**


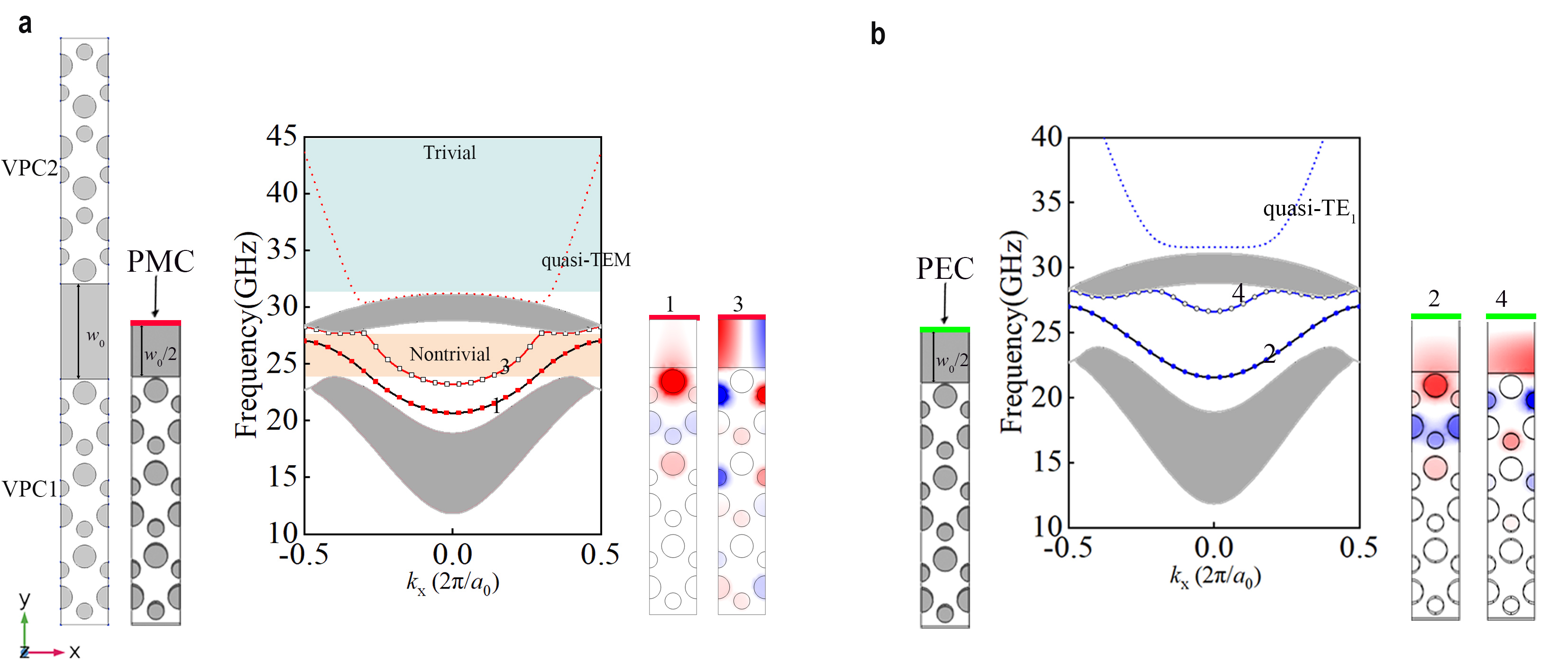

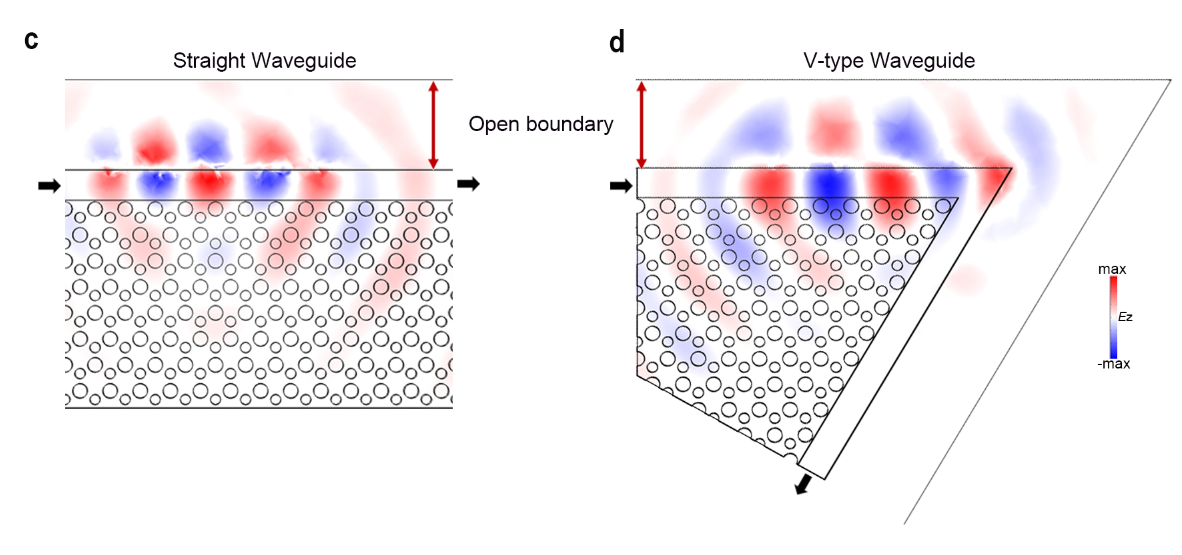


**Figure S5**Ⅰ(a-b) Schematic diagram of the half-supermode VRGW with PMC/PEC boundary and its mode field distribution. (c-d) The half-supermode 1 *E*z distributions under open boundary conditions in straight and V-type waveguides.

In the structural design process of VRGW (**Figure 3**), introducing PEC or PMC boundaries along the ridge center can effectively separate the even- and odd- symmetric modes. **Figures S5(a, b)** present the specific results: when the PMC boundary is applied, even-symmetric modes (supermodes 1, 3, and quasi-TEM modes) are selectively retained (**Figure S5(a)**); when the PEC boundary is applied, odd-symmetric modes (supermodes 2, 4, and quasi-TE_10_ modes) are selectively retained (**Figure S5(b)**). Further field distribution analysis reveals that the half-supermode distribution with PEC/PMC boundaries is the same as the full-supermode distribution state, which indicates that the reduction in structure size does not alter the mode characteristics.

**Figures S5(c-d)** present the transmitted field distributions of half-supermode 1 under open boundary conditions. On the cross-section corresponding to $z > h₁ + g₀$, a clear radiative leakage of the *E*z is observed, which is particularly pronounced in the bend waveguide.

# **S5. VPC1|VPC2 interface combined ridge waveguide**


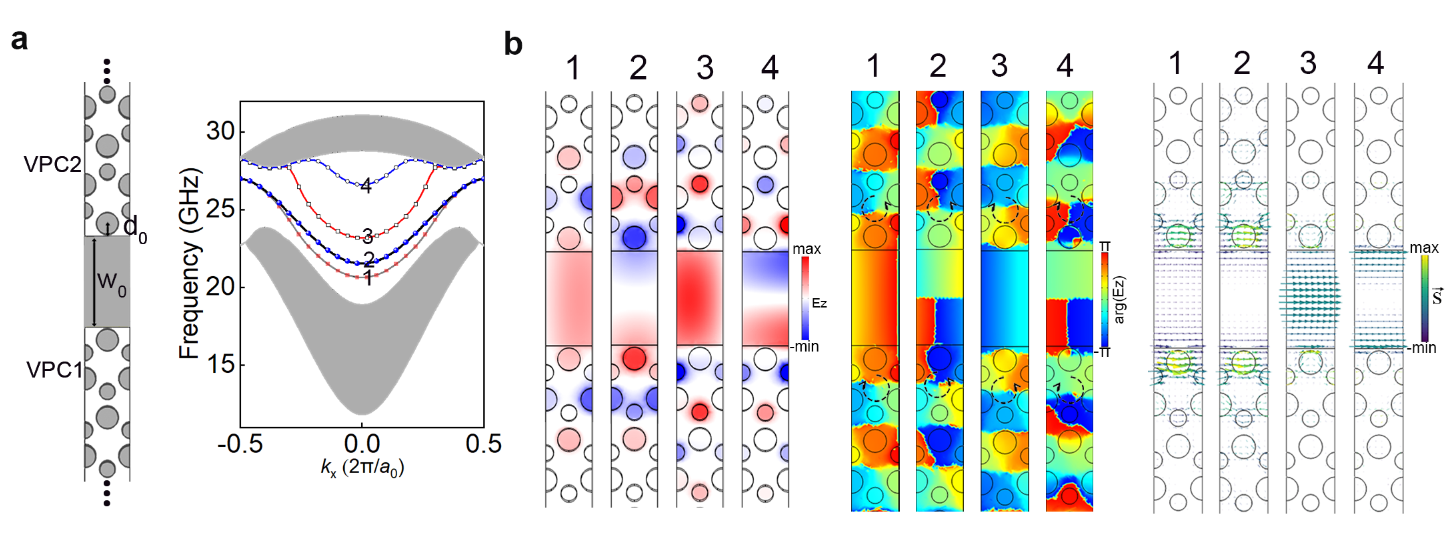


**Figure S6**ⅠModal characteristics in a valley-ridge structure. (a) Schematic of the structure and its dispersion cuver; (b) Field, phase, and Poynting vector distributions of the four characteristic modes.

Here, we systematically analyzed the four supermodes in the valley-ridge structure (**Figure S6(a)**) through their electric-field ($Ez$), phase (arg($Ez)$) and Poynting vector ($S$) distributions (**Figure S6(b)**). All four supermodes exhibit $Ez$ components distributed across both the VPC and ridge regions, with valley vortex phase features in the VPC area.

Supermodes 1 and 2 demonstrate phase continuity at the VPC-ridge interface, indicating a constructive interference between parent modes. As a result, well-hybridized modes are formed. In contrast, supermodes 3 and 4 show phase discontinuity at the interface, implying a lack of constructive interference. Therefore, they become poorly coupled states that retain much of the individual characters of their parent modes. The distribution of the Poynting vector further reveals that supermodes 3 and 4 possess weaker topological vortex characteristics. These features provide visual evidence for understanding the formation mechanism of supermodes.

.

# **S6. VPC2|VPC1 interface combined ridge waveguide (RW)**


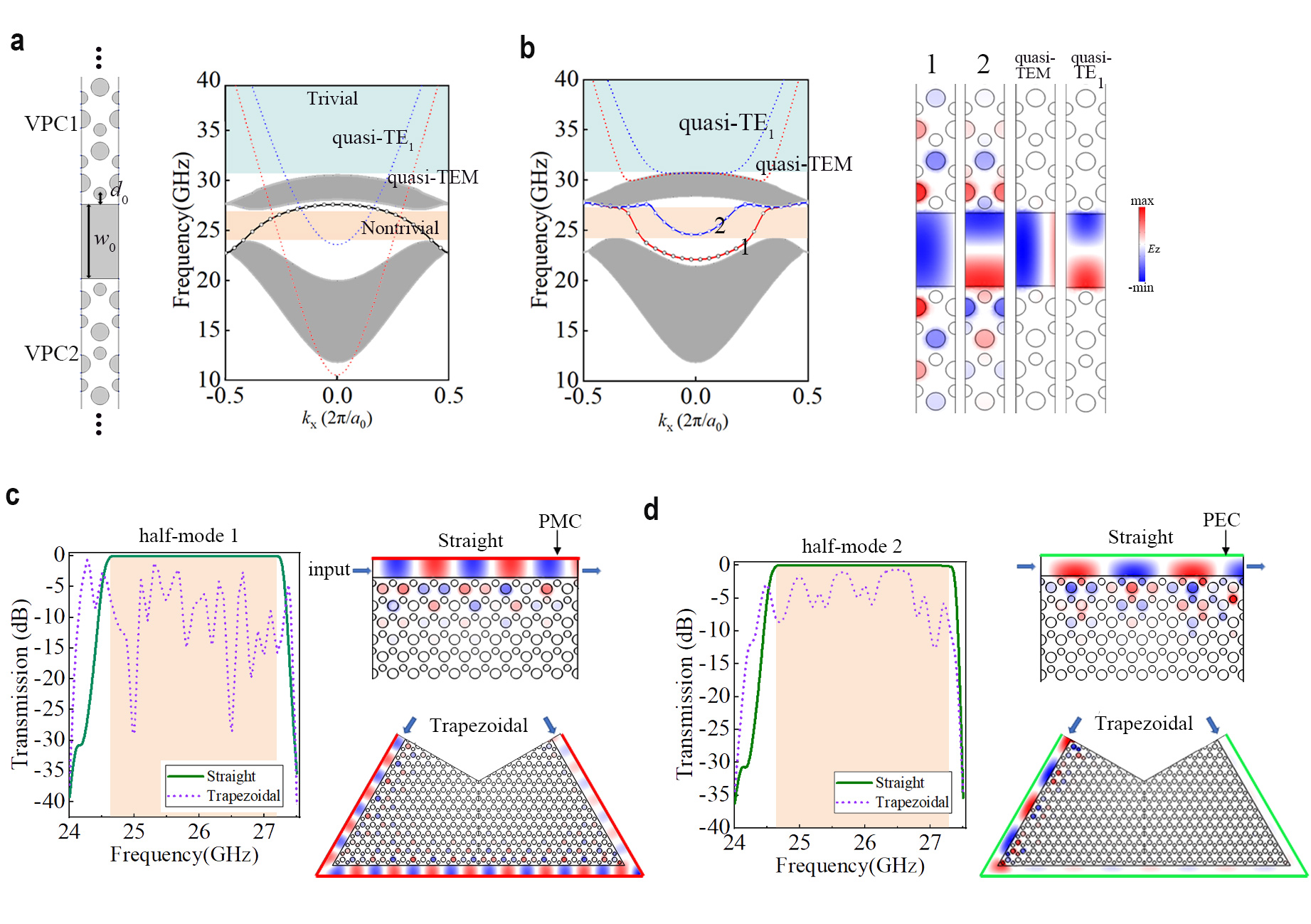


**Figure S7**Ⅰ(a) (Left) Schematic of VPC2|VPC1 interface combined ridge waveguide. (Right) Original band structures of the ridge waveguide and VPC2|VPC1 interface: Quasi-TEM mode (red curve), Quasi-TE_1_ mode (blue curve), and valley edge state (black curve). (b) Band structure and *E*z distribution in the combined waveguide of (a). (c, d) Two half-mode structures constructed using PMC/PEC boundaries. The transmission efficiency and field distributions of half-mode 1 and mode 2 across straight and trapezoidal paths. Parameters: *w*_0_ = 3.5*l*, g_0_ = 0.38 mm, *d*_0_ = *l*/2.

**Figure S7(a)** shows the original band structures of the VPC2|VPC1 domain wall and ridge waveguide. The VPC2|VPC1 interface supports concave-shape valley kink states (solid curve). Since the ridge waveguide exhibits exclusively convex mode dispersion (quasi-TEM, quasi-TE_10_, dashed curves), its modes cannot couple to the valley kink states of the VPC2|VPC1 interface. Consequently, the VPC2|VPC1 structure acts as a conventional periodic cladding, forming a stopband for the conventional gap waveguide. As shown in **Figure S7(b)**, the combined waveguide retains the quasi-TEM and quasi-TE_10_ modes of the ridge waveguide within the trivial bandgap. Within the nontrivial bandgap, their frequencies are redistributed and are referred to as modes 1 and 2, respectively.

To verify the single mode 1 or 2 transmission characteristics, we introduce the PMC/PEC boundary conditions to build a straight and trapezoidal (two 120$^{\circ}$bend paths) half-mode structure, as shown in **Figures S7(c, d)**. The results show that half-modes 1 and 2 suffer significant energy loss during bending, which is similar to the loss of conventional gap waveguides at the bending part. This phenomenon indicates that these two modes maintain the conventional mode characteristics and not protected by the topological phase.

# **S7. Stepped transition rectangular waveguide**


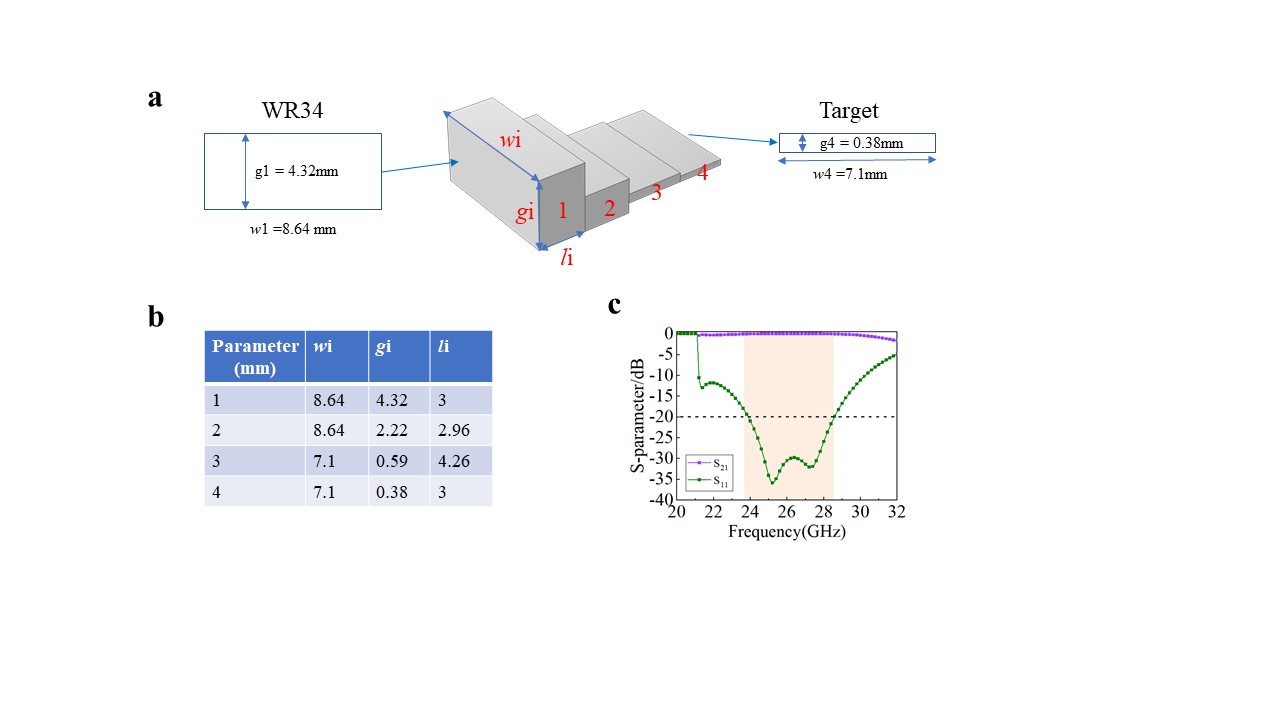


**Figure S8**Ⅰ**Parameter index of** **stepped transition rectangular waveguide.** (a) Stepped transition structure between the initial and the target waveguide port. (b) Details of the parameters of the stepped transition structure. (c) The variation of the S-parameters of the stepped transition rectangular waveguide in the nontrivial bandgap, the S_11_ < -20 dB in the range of 23.5 – 28.3GHz.

# **S8. Transmission characteristics of conventional waveguide**


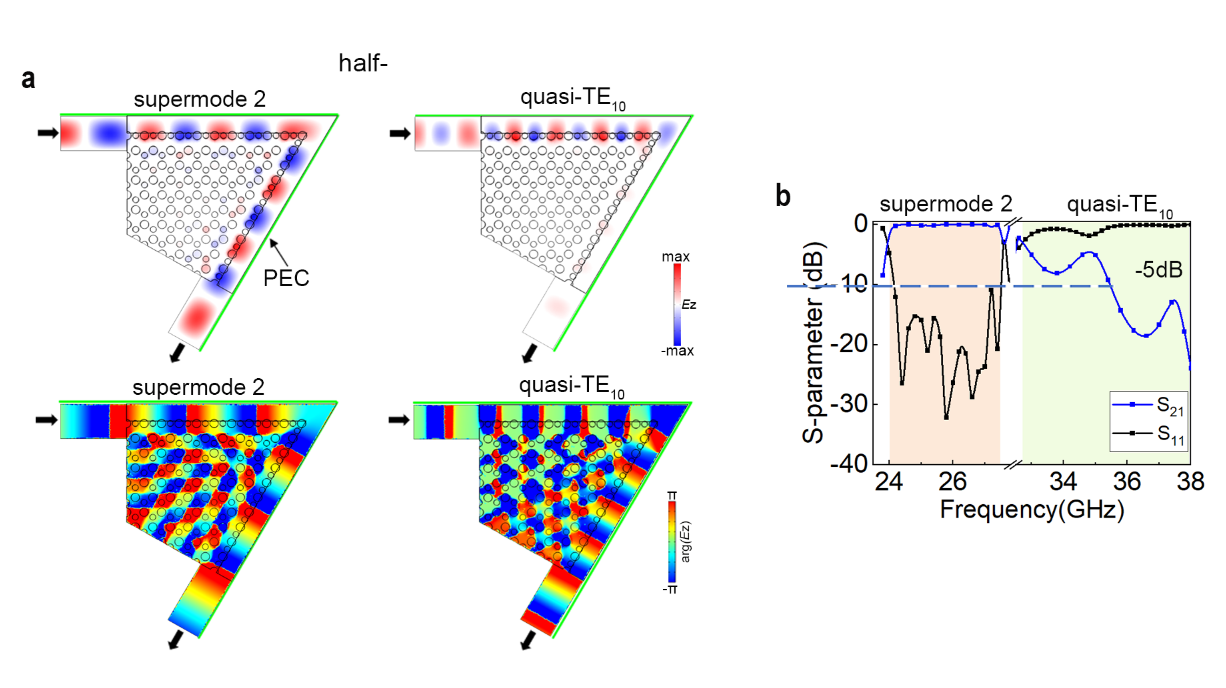


**Figure S9**ⅠTransmission comparison of half- supermode 2 and quasi-TE₁₀ mode (after tuning) in a bend waveguide: *E*z and phase distributions (a) and S-parameters(b).

Here, we have discussion the transmission characteristics of the tuned half-supermode 2 and the conventional half-quasi-TE_10_ mode, as shown in **Figure S9**. The results demonstrate that under bend waveguide, the half-supermode 2 consistently maintains the integrity of its vortex phase feature, whereas the conventional half-quasi-TE_10_ mode exhibits phase distortion. Corresponding S-parameters further confirm that half-supermode 2 nearly lossless transmission (S_21_ > -0.1 dB), while half-quasi-TE_10_ mode transmission loss exceeding -5 dB. This comparison visually confirms the robust signal transmission enabled by topological phase protection.

# **S9.** **Original signal power of USRP ports**


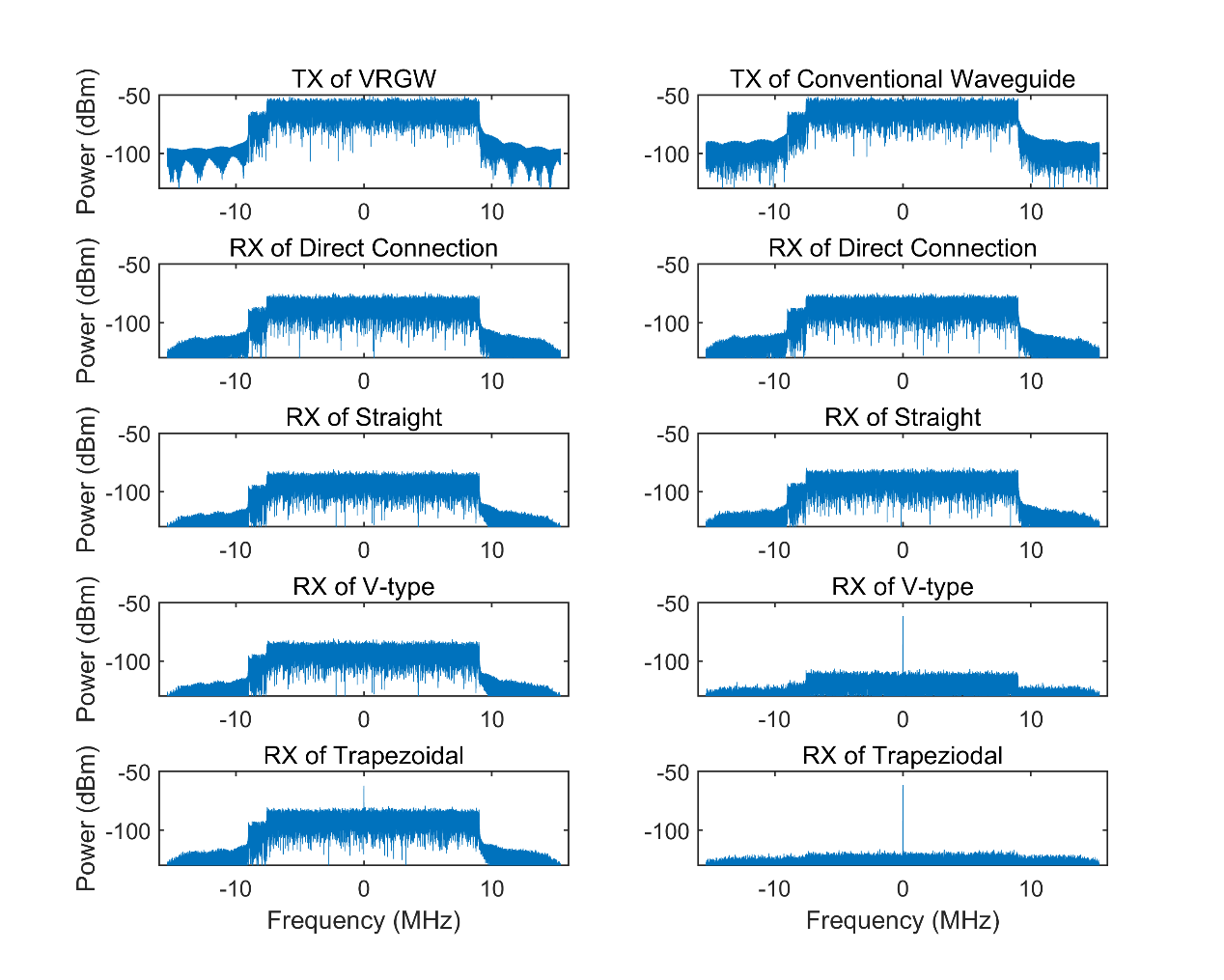


**Figure S10**ⅠOriginal signal power recorded by the USRP.

# **References**

[S1] X-D. Chen, et al. “Valley-contrasting physics in all-dielectric photonic crystals: orbital angular momentum and topological propagation”. *Phys. Rev. B* 96, 020202 (2017).

[S2] A.U. Zaman and P. S. Kildal “Gap Waveguides”. In: Chen, Z. (eds) Handbook of Antenna Technologies. Springer, Singapore (2015).
